# Analysis of the 2016–2018 fluid-injection induced seismicity in the High Agri Valley (Southern Italy) from improved detections using template matching

**DOI:** 10.1038/s41598-021-00047-6

**Published:** 2021-10-19

**Authors:** T. A. Stabile, Josef Vlček, Milosz Wcisło, Vincenzo Serlenga

**Affiliations:** 1 National Research Council, Institute of Methodologies for Environmental Analysis, Tito Scalo, Italy; 2 Institute of Rock Structure and Mechanics, Czech Academy of Sciences, Prague, Czech Republic; 3 Faculty of Mathematics and Physics, Charles University, Prague, Czech Republic

**Keywords:** Petrol, Applied physics, Seismology, Tectonics

## Abstract

Improving the capability of seismic network to detect weak seismic events is one of the timeless challenges in seismology: the greater is the number of detected and locatable seismic events, the greater insights on the mechanisms responsible for seismic activation may be gained. Here we implement and apply a single-station template matching algorithm to detect events belonging to the fluid-injection induced seismicity cluster located in the High Agri Valley, Southern Italy, using the continuous seismic data stream of the closest station of the INSIEME network. To take into account the diversity of waveforms, albeit belonging to the same seismic cluster, eight different master templates were adopted. Afterwards, using all the stations of the network, we provide a seismic catalogue consisting of 196 located earthquakes, in the magnitude range − 1.2 ≤ *Ml* ≤ 1.2, with a completeness magnitude *Mc* = − 0.5 ± 0.1. This rich seismic catalogue allows us to describe the damage zone of a SW dipping fault, characterized by a variety of fractures critically stressed in the dip range between ~ 45° and ~ 75°. The time-evolution of seismicity clearly shows seismic swarm distribution characteristics with many events of similar magnitude, and the seismicity well correlates with injection operational parameters (i.e. injected volumes and injection pressures).

## Introduction

During the last 20 years a variety of conventional and unconventional underground energy projects have been developed to meet the growing energy demand due to the economic development and the rapid increase of world population. Unfortunately, such energy projects can be responsible for induced seismicity which culminated in several cases of damaging earthquakes (e.g.^1–6^). The analysis of induced microseismicity prior to the occurrence of potential damaging earthquake is fundamental not only to provide insights into the physical processes governing induced seismicity, but also for accurate reservoir characterization even at small scale^7,8^ and for the implementation of real-time Adaptive Traffic Light Systems (e.g.^9,10^) for risk management.

The deployment of high-density microseismic monitoring networks has been demonstrated to be a powerful approach to improve the detection performance of weak events^11–18^. In addition to the high density of stations, the main advantages of modern microseismic monitoring networks are: (1) the use of high quality sensors with a large dynamic range placed in shallow or deep boreholes which reduces the background noise level^18–20^; (2) the availability of continuous data streams from each seismic station allowing for the real-time or off-line application of advanced detection^21–23^ and location^24–30^ techniques, which results in decreased magnitude of completeness and generation of massive microseismic catalogues of accurate located events.

**Figure 1:**
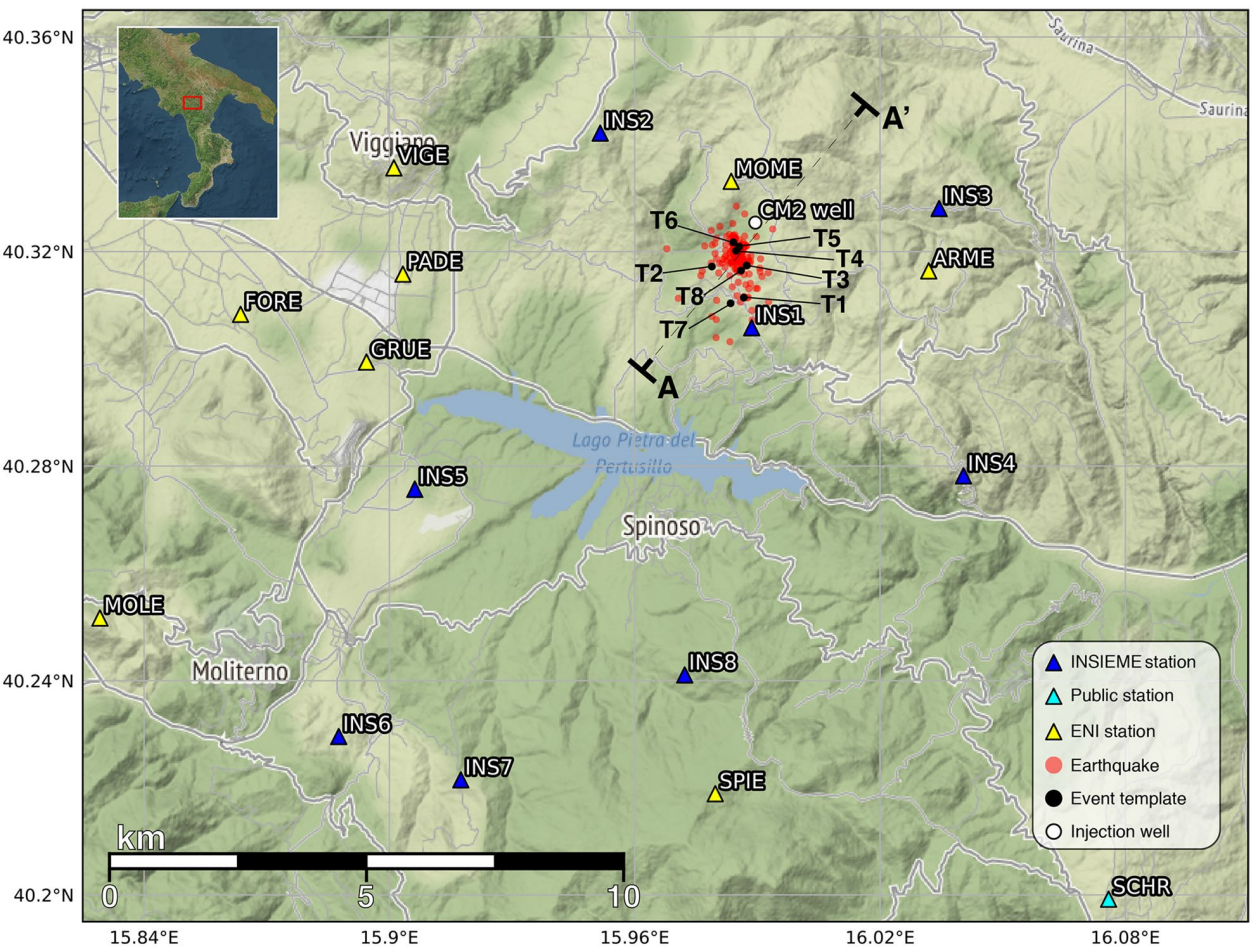
Location in the High Agri Valley (southern Italy) of the fluid injection induced seismicity cluster (red circles) analyzed in this study. Master events (T1–T8) of Supplementary Table S1 are displayed with black circles. Blue triangles represent the stations of the INSIEME seismic network; other public and private stations are represented with cyan and yellow triangles, respectively. The Costa Molina 2 (CM2) injection well is displayed with a white circle. AA′ indicates the trace of the vertical cross section reported in Figs. 4 and 6. [The maps have been generated using Cartopy (https://scitools.org.uk/cartopy/docs/latest/) and Matplotlib (https://matplotlib.org)].

This study is focused on the application of a single-station template matching algorithm based on the cross-correlation technique proposed by Roberts et al.^31^. The use of cross-correlation between a pair of events is a powerful tool in modern seismology to detect small events that can be easily missed by conventional phase arrival‐based methods^32–35^. The advantage in the reduction in magnitude detection thresholds is particularly significant in relation to standard STA/LTA techniques^33,36,37^. We aim to increase the number of detected events belonging to the cluster of fluid-injection induced microseismicity (*Ml* ≤ 2.0)^38^ related to the wastewater disposal activity at the Costa Molina 2 injection well (hereinafter CM2 well, indicated with a white circle in Fig. 1). The injection well is located in the High Agri Valley (Southern Italy) and belongs to the Val d’Agri oilfield, the largest productive on-shore oil field in West Europe that produces hydrocarbons (oil and gas) from a fractured carbonate reservoir. The fluid-induced microseismicity has been identified and studied for the period ranging from 2006 to 2012–2014 by different authors^38–43^ who used triggered data of the seismic network deployed in the study area since July 2001 by the Eni oil company which is managing the Val d’Agri oil filed^44^. In 2016 the INSIEME temporary seismic network^45^ was deployed in the area to study induced seismicity in the framework of the INSIEME project of the Italian SIR-MIUR programme^18^, providing continuous data streams from 8 surface borehole stations. Since 2016 these data allow the detection and location of weak events that were not included neither in the Italian National Seismic Network, nor in the Eni oil company seismic catalogues. The decrease of the completeness magnitude allows us to better study the spatiotemporal evolution of the induced seismicity cluster and its relation with injection operations in the investigation period from 2016-10-12 to 2018-08-31 (about 2 years), when both seismicity recordings and fluid-injection data are available.

## Results

### Basic statistics on event detection using INS1 station

The detection of events was performed by cross-correlating 8 master events (see Supplementary Table S1 online) and continuous data acquired by INS1 station, the nearest station to the analyzed seismicity cluster (see Fig. 1) and the only one installed in a borehole at a depth of 50 m (the others are at 6 m depth), equipped with a 120 s–100 Hz broadband sensor which provides good quality data with low background noise level^18^. In comparison with INS1, the other stations provide disproportionally low signal-to-noise ratio (SNR) of seismic arrivals and do not provide a reliable way of detecting weak events. We set the detection threshold of cross-correlation coefficient *XC* equal to 0.6, a relatively low value for single station detection as we wanted to maximize the number of detected events for further analysis. Waveform examples of true and false detections for *XC* values of 0.65 and 0.70 are displayed in Supplementary Fig. S1. The number of total and true detections (with their percentage reported in parenthesis) for each master is shown in Supplementary Table S2. All masters provided 2002 unique detections out of which 257—roughly 13%—were true detections (comparing to 48 events detected by the STA/LTA technique). Low share of true detections is often tied to low detection threshold, nevertheless, the selection of master events also have effect on the quality of the initial detection catalogue. During the detection process we want to obtain the highest possible number of the true detections with only a limited number of signals that do not include seismic wave arrivals. Addition of further master event is beneficial if it either allows the detection of more unique events or if the values of *XC* of the detected events are higher than in case of other masters. In our case the most problematic master event regarding detection efficiency is template no. 7. While using it allowed us to detect 17 events that were not detected by any other master, it also provided us 1355 false detections which is almost 78% of all false detections. It shows the importance of the master event selection particularly if limited resources are available to perform the analysis. High share of false detections could be also caused by dominance of high amplitude phases within the templates’ waveforms^35,46^. This issue could be minimized by applying the multisegment cross-correlation approach introduced by Gao and Kao^35^ if the optimization of the template matching method is the main goal of the research, particularly when one wants to implement a real-time detection algorithm with a negligible percentage of false alarms. Another aspect of the proper master event selection is the uniqueness of event detection by a given master (see Supplementary Table S3 online). In our catalogue almost 68% of all true events were detected only by either 1 or 2 masters which indicate that we correctly selected masters with varying signal, allowing detection of a broad number of events.

**Figure 2:**
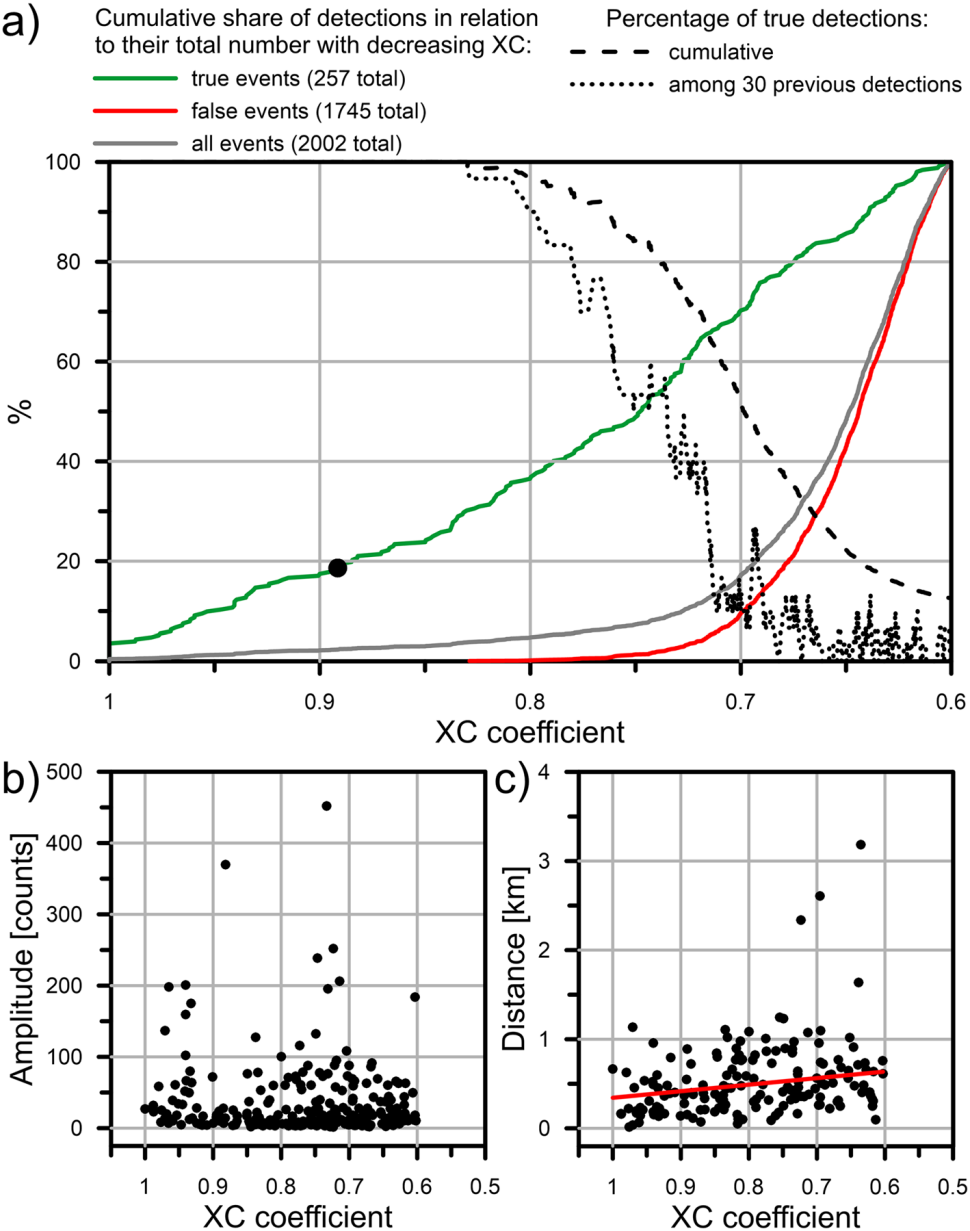
(**a**) Solid lines: share of detections in relation to their total number for *XC* thresholds between 1 and 0.6. for groups including all the 2002 detections (grey line), the 257 true detections only (green line) and the 1745 false detections only (red line). Dashed black line: share of true detections for all detections with *XC* > a given threshold value. Dotted black line: share of true detections within group of consecutive 30 detections with the value of *XC* ≥ a given threshold value. Black dot indicates the share of detections obtained with STA/LTA technique in relation to amount of true events detected with cross-correlation. (**b**) Amplitudes of the detected true events as a function of *XC* coefficient (highest value of *XC* is considered if more than 1 master detected a given event). (**c**) Epicentral distance between located events of the cluster and the master with the highest value of the *XC* coefficient against the *XC* value. The red line represents the linear regression of the distribution obtained by neglecting the 4 farthest events.

The balance between obtaining as complete catalogue as possible and the time necessary for the analysis depends significantly on the *XC* threshold set for detections. Low *XC* threshold set in the detection process that allowed us to detect high number of small events, gives us a possibility to briefly illustrate how the catalogue changes with increasing *XC* threshold. Figure 2a summarizes how the share of the true, false and all detections changes in relation to their total number with decreasing threshold of *XC* from 1 to 0.6. We can see that a few first false detections are in the *XC* value range 1–0.8 where almost twice the number of the true events were detected when compared to results of STA/LTA techniques (48 events, black dot in Fig. 2a). Changing the threshold to 0.7 would provide us ~ 70% of true detections obtained by using the threshold equal 0.6, while reducing false detections by ~ 90%. Share of true detections in the *XC* > 0.7 interval is close to 50%. Naturally, locally, the share of true detections decreases below 50% earlier. Summarizing, it is clear that when limited resources are available, the use of cross-correlation with higher detection threshold is still very attractive method providing a good catalogue completeness.

It is also worth to note that, in case of standard STA/LTA technique, high SNR of arrivals is required. In Fig. 2b we can see that high values of cross-correlation are not necessarily connected to high values of amplitudes of the detected true events. Most of the detected events are in fact of low amplitude. These events are not detectable by using standard STA/LTA techniques.

Cross-correlation detection is a robust technique if we deal with clustered seismicity. Distant events are not as likely detected by the *XC*. Result of the differences in location of events belonging to the cluster against the value of cross-correlation between waveforms is shown in Fig. 2c. We can see that there is a mild trend, with events detected with lower value of the *XC* being located on average further away from the master. The trend is not particularly strong if compared with general variability (likely caused by the low SNR of detected events), still it is worth to note that all the 4 farthest events were detected with relatively low value of *XC*. It shows that in case of bigger clusters, selection of masters located in different areas is necessary.

### Event location, frequency-magnitude distribution and completeness magnitude

196 out of 278 events (see “Methods” section for details) were relocated by applying the inversion procedure and constraints described in the “Methods” section. Among the 82 excluded events, 54 were outliers (located outside the cluster) and 28 were considered detectable as they were recorded only by INS1 station (i.e., the reference station used for template matching detection) or at most also by INS3 station. High-precision hypocenter relocations were determined with relative location errors ranging between 13 and 154 m in the horizontal and depth directions, except for two events that have relative location errors ranging between 127 and 589 m. Supplementary Fig. S2 shows the projection of relocated events along the vertical cross section of the AA′ profile displayed in Fig. 1 with their horizontal and vertical relative location errors. The largest RMS residual of 0.056 s was observed at SIRI station (http://terremoti.ingv.it/en/instruments/station/SIRI) of the Italian National Seismic Network^47^.

The seismicity cluster is included in a volume with latitude from 40.30 °N to 40.33 °N, longitude from 15.96 °E to 16.00 °E, and from 3.15 to 4.66 km depth below sea level. Relocated events of the cluster have local magnitude estimates − 1.2 ≤ *Ml* ≤ 1.2, thus applying Eq. (5) (see “Methods” section) their moment magnitude estimates vary between − 0.2 ≤ *Mw* ≤ 1.5. The supplementary seismic catalogue (see file “DataS1.csv” online) lists the hypocentral parameters, magnitude and seismic moment estimates of all the located events; for detected events only the date and detection time are reported.

**Figure 3:**
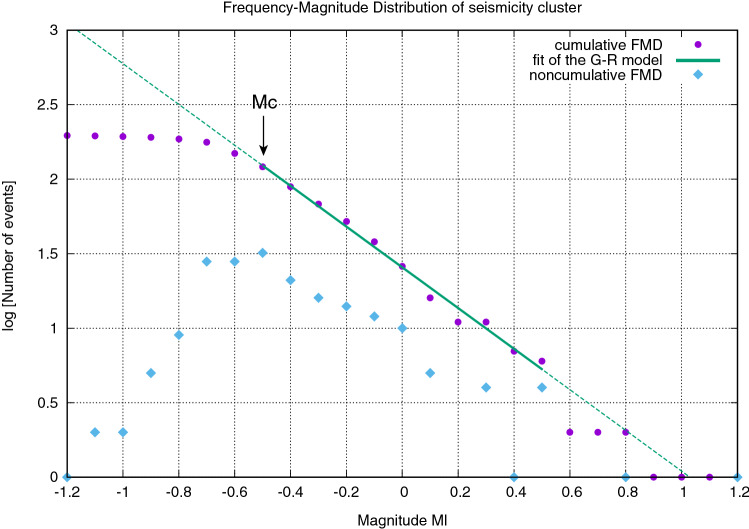
The frequency–magnitude distribution (FMD) of the analyzed induced seismicity cluster. The green line indicates the fit of the Gutenberg-Richter model over the local magnitude range *Mc* ≤ *Ml* ≤ 0.5, where *Mc* = − 0.5 is the completeness magnitude. The dashed green line is the extrapolation of the Gutenberg-Richter model for the higher and the lower local magnitude values. Circles and diamonds represent the cumulative and the noncumulative FMD, respectively.

The completeness magnitude *Mc* of the seismicity catalogue was computed from the frequency-magnitude distribution (FMD) of seismicity with magnitude step size of 0.1 by applying the maximum curvature method^48^ on the non-cumulative FMD (cyan diamonds in Fig. 3) and the entire magnitude range method on the cumulative FMD (violet circles in Fig. 3). Both methods provided the same estimate of *Mc* = − 0.5 ± 0.1, which is much smaller than the completeness magnitude of the Eni catalogue (*Mc* = 1.1 ± 0.1)^38,39^ and of the Italian National Seismic Network in the HAV (*Mc* ~ 1.5)^49^; indeed, in the same period of observation, only the largest magnitude event of the cluster (*Ml* = 1.2 of 2018-01-29 at 15:23:11 UTC time) was reported in the Italian National Seismic Network catalogue and only 8 events (6 located, including the three strongest events, and 2 only detected) of the seismicity cluster were reported in the Eni catalogue^50^. Considering only events with *Ml* ≥ *Mc* and with *Ml* ≤ 0.5 (only two events have *Ml* > 0.5), the parameters of the Gutenberg–Richter model (log_10_*N* = *a* − *b Ml*, with *N* as the number of events having magnitude ≥ *Ml*; green line in Fig. 3) were inferred by applying the nonlinear Levenberg–Marquardt least-squares algorithm^51^, thereby obtaining *a* = 1.41 ± 0.01 and *b* = 1.37 ± 0.05 with a residual sum of squares *RSS* = 0.02 and a coefficient of determination *R*^2^ = 0.99.

### Spatiotemporal evolution of seismicity and its relation with injection operations

**Figure 4:**
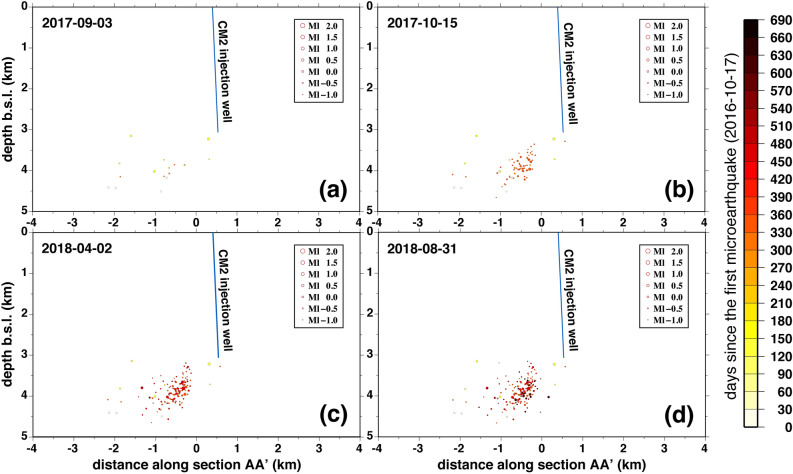
Projection of microearthquake hypocenters (only events relocated with double-difference method) along the vertical cross section of the AA′ profile displayed in Fig. 1 in four different time periods: (**a**) until 2017-09-03; (**b**) from 2017-09-17 to 2017-10-15; (**c**) from 2017-12-31 to 2018-04-02; (**d**) from 2018-04-14 to 2018-08-31. The dimension of the circles is proportional to the local magnitude of the seismic events whereas the color of the circles indicates the earthquake origin time expressed in days since the first event. The blue line in each panel represents the projection of the CM2 injection well along the AA′ vertical cross section.

The seismicity cluster is characterized by a swarm type time-dependent earthquake occurrence with many events of about the same magnitude. We can distinguish four periods of time where the seismicity shows different space–time characteristics. The first one from 2016-10-12 to 2017-09-03 (Fig. 4a) is characterized by a low seismicity rate with 20 located events (and further six detected) occurred in about 10 months. These events are already widespread in the entire volume occupied by the full seismicity cluster; in this period, the maximum local magnitude of seismicity is *Ml* = 0.5 (*Mw* = 1.0). The second period, which lasted until 2017-10-15 (Fig. 4b) is characterized by the highest seismicity rate with 65 located events (and further 7 detected) occurred in only 1 month (from 2017-09-17 to 2017-10-15), out of which 13 occurred on 2017-09-30; in this period, the maximum local magnitude of seismicity is *Ml* = 0.8 (*Mw* = 1.3). The third period, which lasted until 2018-04-02 (Fig. 4c) is also characterized by high seismicity rate, but lower than in the previous period, with 68 located events (and further 8 detected) occurred in about 3 months; in this period the largest magnitude event (*Ml* = 1.2; *Mw* = 1.5) occurred. Finally, the fourth period (Fig. 4d) is characterized by a seismicity rate lower than the rate observed in the second and the third period with 43 located events (and further 7 detected) occurred in about 5 months, the 20% of them gathered in only two days (second and third July 2018); in this period, the maximum local magnitude of seismicity is again *Ml* = 0.5 (*Mw* = 1.0). Except for the first period, the seismicity located from the second to the fourth period, principally occurred within 2 km distance from CM2 injection well and seems to mainly illuminate the NW–SE trending damage zone. This supports the presence of SW-dipping fault proposed by Buttinelli et al.^40^ based on the interpretation of reflection data from the 3D survey provided by Eni Company, and modelled by Vadacca et al.^52^.

**Figure 5:**
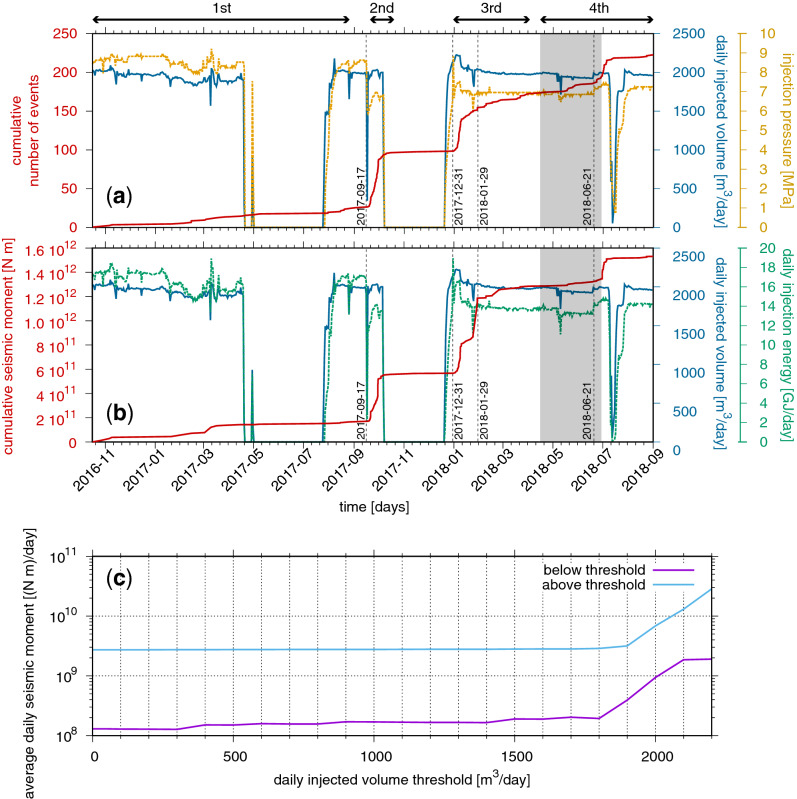
(**a**,**b**) Wellhead operational parameters against the cumulative number of induced events (including those only detected) and the cumulative seismic moment of located events during time. (**a**) Cumulative number of events (solid red line) against the average fluid injection pressure (dashed yellow line) and the daily injected volumes (solid blue line). (**b**) Cumulative seismic moment of events (solid red line) against the daily fluid injection energy (dashed green line) and again the daily injected volumes (solid blue line). The four periods of time where the seismicity shows different space–time characteristics are indicated on the top of the panels by means of black double-ended arrows. Crucial dates are indicated with vertical dashed grey lines. (**c**) Average daily seismic moment of events occurred above (cyan line) and below (violet line) a given injection rate threshold as a function of the daily injection rate threshold.

The underlying driving mechanism governing the time-dependent variation of seismicity rate is better understood if the time evolution of seismicity is compared with the time series of wellhead injection operational parameters. Figure 5a shows the comparison between the cumulative number of detected and located events (continuous red line) with the daily injected volumes *V* (continuous blue line) and the daily average injection pressure *P* (dashed yellow line), whereas Fig. 5b shows the comparison between the cumulative seismic moment of located seismicity (continuous red line) with the daily injection energy *E*_*inj*_ = *PV* (dashed green line) and again the daily injected volumes (continuous blue line). It is possible to observe from operational parameters that injection operations were suspended for about 3 months from 2017-04-21 to 2017-07-25 (except for 30 April and 1 May 2017), for 74 days from 2017-10-08 to 2017-12-20, and on 14 and 15 July 2018. During these periods only 8 events were detected (7 of them also located; their local magnitude is within − 1.1 ≤ *Ml* ≤ − 0.2); 7 out of 8 events occurred within 8 days from the stop of injection operations. Furthermore, in the first period from 2016-10-12 to 2017-08-26 the few seismic events (Fig. 4a) principally occur when rapid changes in volumes and pressures (and thus, the energy) are applied (Fig. 5). The second period of seismicity from 2017-09-17 to 2017-10-15 (Fig. 4b) is characterized by the highest seismicity rate which is enhanced by acidification operations carried out by the Eni Company on 17 September 2017. The goal of the operation was the removal of the plugging material in order to re-establish and preserve the injectivity of the reservoir. This is clear by looking at the time series of the daily average injection pressure in Fig. 5a, which was reduced from an average value of 8.5 MPa to an average value of 7 MPa after such operation. The swarm lasts for about 1 month until the injection was suspended. The onset of the third period of seismicity (Fig. 4c) is observed 10 days after the restart of injection operations and in correspondence to the day (2017-12-31) when a rapid increase of the daily average injection pressure from about 6.5 MPa to about 8.8 MPa is applied. During the first week of 2018 both the daily injected volumes (2180 ≤ *V* ≤ 2225 m^3^/day) and average injection pressure (7.15 ≤ *P* ≤ 7.45 MPa) were above the average values applied after the acidification operation (~ 2000 m^3^/day and ~ 7 MPa, respectively). In these days the seismicity rate is comparable to that observed during the second period of seismic activity (Fig. 5). Afterwards, the seismicity rate gradually decreases even if the largest event of the cluster occurs on 2018-01-29 (Figs. 4c and 5b). Such decrease of seismicity rate fits with the contemporary reduction of the daily average pressure to ~ 7 MPa from 9 January 2018 and the gradual decrease of the daily injected volumes from 2120 m^3^/day on 9 January 2018 to 1995 m^3^/day on 14 February 2018. At the beginning of the fourth period, more specifically from 2018-04-14 to 2018-06-29 (grey area in Fig. 5), the station INS1 suffered a significant disturbance and the station INS3 was used for detection (see “Methods” section for details). This allowed us to not lose information about fluid-induced microseismicity and to observe the increase of seismic events corresponding to the slightly increase of injection energy (increase of both injected volumes and injection pressure) operated on 21 June 2018 (Fig. 5).

## Discussion

Our method for detecting weak microseismic events is based on a single-station template matching algorithm which evaluates the waveform similarity of the continuous data stream with selected master events through the cross-correlation coefficient *XC*. We apply the algorithm to the recordings of fluid-injection induced seismicity cluster close to the CM2 injection well in the High Agri Valley in the period from 2016-10-12 to 2018-08-31. Because our goal is to detect as many events as possible, the threshold is fixed to *XC* = 0.6 even if we observe the share of true detections not exceeding 10% for any 30 consecutively ranked detections up to the value of *XC* ~ 0.67 (Fig. 2a). Also the use of several event templates is aimed to enhance the detectability of microearthquakes whose waveforms can differ from each other despite the seismic swarm is strongly clustered (Fig. 1). Indeed, the use of a poor number of master events may lead to some slave events being omitted because each master template is able to detect exclusive events (see Supplementary Table S2 online). In this way we detected 224 events belonging to the seismicity cluster, with 196 of them also located. The events of the cluster have local magnitude estimates − 1.2 ≤ *Ml* ≤ 1.2 and the completeness magnitude is *Mc* = − 0.5, much lower than the completeness magnitude obtained for such seismicity cluster in previous studies (*Mc* = 1.1)^38,39^. The relative high b-value of 1.37 ± 0.05, inferred from the frequency-magnitude distribution of seismicity (Fig. 3), is likely to suggest the diffusion of pore fluid pressure as the underlying physical driving mechanism of observed seismicity^53^. This is also supported by the swarm-type distribution of the analyzed seismicity cluster with no identifiable mainshock (see Supplementary file DataS1.csv online and Fig. 5).

**Figure 6:**
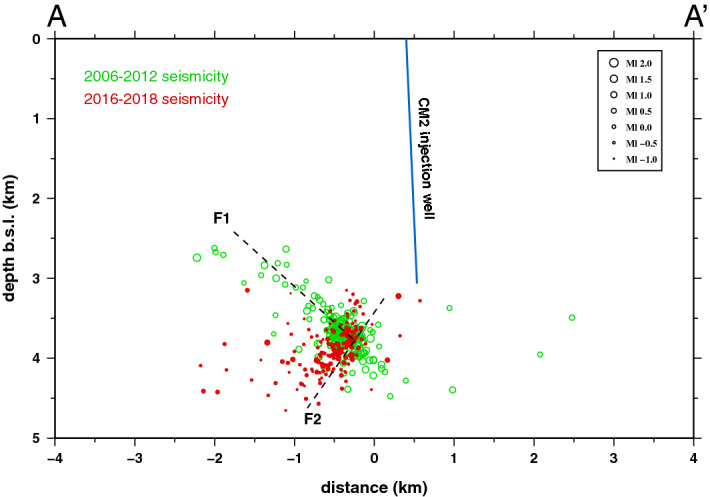
Projection of microearthquake hypocenters of the 2006–2012 seismicity (green circles) and the 2016–2018 seismicity relocated with double-difference method (red circles) along the vertical cross section of the AA′ profile displayed on the map of Fig. 1. The dimension of circles is proportional to the local magnitude of the seismic events. The blue line represents the projection of the CM2 injection well along the AA′ vertical cross section. Dashed lines represent the projection along the AA′ vertical cross section of the NW-trending, NE-dipping back-thrust reactivated by fluid injection operations (F1) and the SW-dipping thrust (F2) acting as hydraulic connection between the injection well and the F1 fault.

The accurate relative location of 170 events by applying the double-difference method^24^, and considering the 3D P- and S-wave velocity model of the area proposed by Serlenga and Stabile^43^, allows depicting the distribution of microearthquakes also along the damage zone of the SW-dipping fault hypothesized by Buttinelli et al.^40^ and modelled by Vadacca et al.^52^ which was not observed before through the seismicity distribution. Figure 6 shows the projection along the AA′ section (see Fig. 1) of the relocated 2006–2012 seismicity (green circles) recorded by the Eni seismic network and analyzed in previous studies^38–43^, and the 2016–2018 seismicity (red circles) located in this study. It is possible to observe that 2006–2012 seismicity is distributed along the NW-trending, NE-dipping back-thrust reactivated by fluid injection operations (F1 fault in Fig. 6) whereas the 2016–2018 seismicity is mainly distributed along the intersection between the NE-dipping back-thrust and the SW-dipping thrust (F2 fault in Fig. 6), the latter acting as hydraulic connection between the injection well and the NE-dipping back-thrust. This hydraulic connection can justify the rapid onset of induced seismicity observed only 3 h after the begin of injection^39^. Vadacca et al.^52^ hypothesized that SW-dipping thrust activates only with an aseismic creeping deformation because no microearthquakes have been observed, but probably microearthquakes along this fault were not observed simply due to the relative high completeness magnitude of seismicity analyzed in previous studies and because the 2016–2018 seismicity has been particularly enhanced by acidification operations.

Because the study area has experienced different stress regimes during its geological history (e.g., see^54^ for Southern Apennines and in particular^55^ for the High Agri Valley), it is likely to hypothesize that rocks may have networks of fractures and faults with different orientations. This also explains why the hypocenters of relocated events are scattered even if location errors are estimated accurately by the singular value decomposition (SVD). Considering the current normal stress regime, we can determine which fractures are critically stressed and on which mis-oriented faults the slip can be activated with a maximum pore pressure perturbation of 6 MPa. This value corresponds to the pore pressure increase computed at the bottom-hole by subtracting the pressure drop of about 3 MPa along the pipe of the injection well (information provided by the Eni Company) from the maximum injection pressure operated by the company in the period of study (see Fig. 5), and neglecting additional pressure drop factors into the reservoir. First, we calculate at the bottom-hole the vertical stress *S*_*V*_, which is the maximum principal stress (*S*_*1*_) in normal faulting regimes, by using the information reported in Supplementary Table S4 online. The obtained estimate of *S*_*V*_ ranges between 9.82 × 10^7^ Pa and 1.06 × 10^8^ Pa depending on the use of the minimum and maximum values of density for each formation. Subsequently, the least principal stress *S3* can be computed through the relationship obtained by Jaeger and Cook^56^ when a critically oriented fault is at the frictional limit:

1 $$\frac{{\sigma_{1} }}{{\sigma_{3} }} = \frac{{S_{1} - P_{p} }}{{S_{3} - P_{p} }} = \frac{{S_{V} - P_{p} }}{{S_{Hmin} - P_{p} }} = [(\mu^{2} + 1)^{1/2} + \mu ]^{2} ,$$

where $\sigma_{1}$ and $\sigma_{3}$ are the maximum and the minimum effective stress, respectively, *P*_*p*_ is the reservoir pore pressure, and $\mu$ is the coefficient of friction.

**Figure 7:**
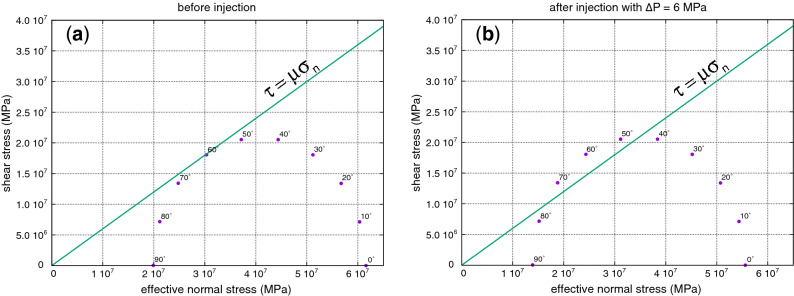
Mohr circle graphical representation of the state of stress on individual planes with $\beta$ angle step size of 10° (violet dots) and its comparison with Mohr–Coulomb shear failure envelope $\tau = \mu \sigma_{n}$ (solid green line) for (**a**) the stress state before fluid injection and (**b**) the stress state after fluid injection with a pore pressure increase at the bottom-hole of $\Delta P_{p} =$ 6 MPa.

For $\mu = 0.6$ we obtain $\frac{{\sigma_{1} }}{{\sigma_{3} }} = 3.1$, thus rearranging the Eq. (1) we obtain:

2 $$S_{Hmin} = \frac{{S_{V} - P_{p} }}{3.1} + P_{p} ,$$

such that considering the estimates of *S*_*V*_ and considering the initial reservoir pressure^38^ *P*_*p*_ = 3.66 × 10^7^ Pa, the estimate of *S*_*Hmin*_ using Eq. (2) ranges between 5.65 × 10^7^ Pa and 5.90 × 10^7^ Pa. Indicating with $\beta$ the angle between the fault normal and $\sigma_{1}$, it is possible to define the shear stress $\tau$ and the effective normal stress $\sigma_{n}$ acting on each fracture as functions of $\beta$, $\sigma_{1}$ and $\sigma_{3}$ (e.g., see^7^). We may then evaluate for which $\beta$ angles fractures are critically stressed by plotting the corresponding Mohr–Coulomb failure envelope for given values of $\sigma_{1}$ and $\sigma_{3}$ (e.g.^7,57^). Figure 7a shows the comparison between the plot of the Mohr circle, with $\beta$ angle step size of 10°, and the Mohr–Coulomb shear failure envelope ($\tau = \mu \sigma_{n}$) for $\sigma_{1}$ and $\sigma_{3}$ computed considering the initial reservoir pressure; Fig. 7b shows the same comparison but considering a pore pressure increase $\Delta P_{p} =$ 6 MPa ($\sigma_{1} = S_{V} - (P_{p} + \Delta P_{p} )$; $\sigma_{3} = S_{Hmin} - (P_{p} + \Delta P_{p} )$). Considering that in the High Agri Valley the maximum principal stress is vertical, it is easy to demonstrate through geometrical considerations that in this case the $\beta$ angle corresponds to the fracture dip. From Fig. 7 one can observe that in the unperturbed medium only fractures that dip ~ 60° from the horizontal are critically stressed, as expected in normal faulting regimes (Fig. 7a), whereas a pore pressure increase of 6 MPa can critically stress fractures that dip from ~ 45° to ~ 75° (Fig. 7b) if additional pressure drops factors into the reservoir are neglected. This finding justifies the observation of significant changeability of waveforms within the set of events belonging to the same cluster: several different sets of fractures and faults that dip from ~ 45° to ~ 75° have been reactivated by fluid injection. In this study we derived also the following simple equation to analytically compute the minimum and maximum $\beta$ angles of critically stressed fractures for given values of $\mu$, $\sigma_{1}$, and $\sigma_{3}$:

3 $$\beta_{min,max} = \tan^{ - 1} \left[ {\frac{{ - 1 \pm \sqrt {1 + \mu^{2} \left( {1 - k^{2} } \right)} }}{{\mu \left( {1 - k} \right)}}} \right]$$

with $k = \left( {\sigma_{1} + \sigma_{3} } \right)/\left( {\sigma_{1} - \sigma_{3} } \right)$. The mathematical derivation of Eq. (3) is given in Supplementary Information online.

The high number of detections obtained through the application of the single-station template matching algorithm allows the statistical investigation of the relationship between the applied injection rates and seismicity. The first key point can be seen in Fig. 5 from the comparison over time between seismicity and operational parameters. In the first period from 2016-10-12 to 2017-09-03, when acidification treatment was not yet executed, only the 12% (26 events out of 224) of the total seismicity of the investigated cluster (detected + located events) was observed. In this period, operations injection rates even of 2000 m^3^/day did not affect significantly the earthquake production. On the other hand, the 88% (198 out of 224) of seismicity was observed after acidification operations carried out on 17 September 2017, thus the statistical population of microearthquakes accounts mainly for those occurred after the acid treatment. Table 1 reports for different injection rate thresholds (column 1): the number of events (only detected + located), the cumulative seismic moment of located events, and the number of injection days below (columns 2–4) and above (columns 5–7) the respective injection rate threshold. Furthermore, columns 8–10 of Table 1 indicate also the percentage out of total of the number of events, the cumulative seismic moment, and injection days above the given threshold. Only 8 events occurred with injection rates equal or less than 100 m^3^/day. The number of events (and their cumulative seismic moment) still remains low with injection rates equal or less than 1900 m^3^/day, whereas it increases when injection rates above 1900 m^3^/day are applied. This finding is well illustrated in Fig. 5c: when fluid injection is operated with injection rate up to 1800 m^3^/day the average daily seismic moments of events occurred above and below the given threshold remain constant to values of about 3 × 10^9^ N m/day and about 2 × 10^8^ N m/day, respectively; conversely, the average daily seismic moment of events occurred above and below the given threshold follows an exponential growth if injection rates greater or equal than 1900 m^3^/day are applied. We can argue that the 2016–2018 fluid-induced microseismicity is mainly activated after acidification operations and for injection rates above 1900 m^3^/day, but never exceeding the magnitude threshold of *M* = 1.5 that triggers the level of attention of the four-level traffic light system introduced in the Italian guidelines^58,59^. When acid treatment is not executed, injection rates of about 2000 m^3^/day do not affect significantly the earthquake production; this is in agreement with rate–state simulations recently provided by Hager et al.^50^.

**Table 1:** Number of events (including those only detected), cumulative seismic moment of located events, and injection days below and above a given injection rate threshold. The last three columns also indicate the percentage of the number of events, the cumulative seismic moment, and the injection days above the given threshold out of the respective totals.

Injection rate threshold (m^3^/day)	N. ev. below	Cum. Mo below (N m)	Inj. days below	N. ev. above	Cum. Mo above (N m)	Inj. days above	N. ev above/Tot. ev (%)	Mo above/Tot. Mo (%)	Inj. days above/Tot. days (%)
100	8	2.20E+10	171	216	1.52E+12	559	96.4	98.6	76.6
1000	11	3.07E+10	181	213	1.52E+12	549	95.1	98.0	75.2
1800	14	3.98E+10	206	210	1.51E+12	524	93.8	97.4	71.8
1900	23	1.07E+11	274	201	1.44E+12	456	89.7	93.1	62.5
2000	104	5.48E+11	584	120	9.99E+11	146	53.6	64.6	20.0
2100	185	1.33E+12	713	39	2.21E+11	17	17.4	14.3	2.3
2200	192	1.38E+12	724	32	1.71E+11	6	14.3	11.1	0.8

Summarizing, the main findings of this study are reported below:

- The single-station template matching algorithm allowed us to detect weak events with local magnitudes in the range − 1.2 ≤ *Ml* ≤ 1.2 and with a completeness magnitude *Mc* = − 0.5, much lower than the completeness magnitude obtained for this seismicity cluster in previous studies.
- The seismicity is characterized by a swarm time-dependent earthquake occurrence with no identifiable mainshock and a *b*-value of 1.37 ± 0.05, inferred from the frequency-magnitude distribution of seismicity (Fig. 3). Furthermore, the seismicity well correlates with injection operational parameters (Fig. 5 and Table 1) with only 8 events occurred with injection rates equal or less than 100 m^3^/day. These observations suggest the reduction of the frictional fault strength due to pore fluids pressure.
- The distribution of hypocenters obtained from relative locations depicts the damage zone of a SW dipping fault (Fig. 4). It may constitute a hydraulic connection path between the bottom-hole of the CM2 well and the NE dipping back-thrust which was originally reactivated by injection operations in the period 2006–2012 (Fig. 6).
- The maximum injection pressure at the bottom-hole of 6 MPa operated by the oil company may critically stress fractures dipping in the range from ~ 45° to ~ 75° (Fig. 7), thus justifying the observed variety in the earthquake waveforms (Supplementary Figs. S3 and S4).
- A strong increase in the number of seismic events is observed after acidification operations and for injection rates greater or equal than 1900 m^3^/day, but never exceeding the magnitude threshold (*M* = 1.5) that activates the attention level introduced in the Italian guidelines. When acid treatment is not executed, injection rates of about 2000 m^3^/day do not significantly affect the earthquake production.

The methodology adopted in this study has been proven to be useful to support the management of the industrial activity and for enhancing the capability of decisional protocols to prevent the occurrence of critical events. On these grounds, a step forward is its implementation into fully automatized procedures, where the selection of event templates, the discrimination between true and false detections, and the automatic picking of P- and S-waves could be based on deep neural network algorithms (e.g., the PhaseNet algorithm^60^ for the seismic arrival time picking) and/or on unsupervised/supervised machine learning approaches (e.g., using the Scikit-learn Python package^61^).

## Methods

### Single-station template matching algorithm

Many data processing methods allow detecting very weak seismic events provided a sufficient station coverage. In this work, we implemented a single-station template matching algorithm based on the technique proposed by Roberts et al.^31^ for the analysis of three-component seismic data from a single station. The nearest station to the analyzed seismicity cluster (INS1 station, see Fig. 1) of the INSIEME seismic network^18^ was used for detection and the eight different master events reported in Supplementary Table S1 were selected to automatically detect weaker signals with high similarity of waveforms. Supplementary Fig. S3 shows the waveforms of the eight selected master event templates T1–T8 in ground velocity and acceleration: it is possible to observe that the waveforms of templates T1–T8 slightly differ from each other for all the components. In particular, P-wave first motion clearly changes both in amplitude and polarity as evinced in Supplementary Fig. S4 where the ground acceleration waveforms of the eight master templates are zoomed around the P-wave arrival.

High number of templates was selected to detect as many new events as possible, given significant changeability of waveforms within the set of events belonging to the same cluster. Different template lengths of 0.6, 0.8 and 1 s were tested in order to check the number of false detections first. Shorter templates generally increase cross-correlation coefficient (*XC*), particularly for false detections, because the shorter is the length, the higher is the probability of anthropogenic noise to have a pattern coherent with the template waveform and the higher is the rate of false events. For these reasons the longest tested template of 1 s was manually selected around the first P-wave arrival on the vertical component (CHZ channel) and around the S-wave arrival on the horizontal components (CH1 and CH2 channels), respectively (signals highlighted in red in Supplementary Fig. S5). The detection threshold of the cross-correlation coefficient was set to 0.6 for all components of the seismograms. Subsequently, all the templates and the continuous data stream of INS1 station were band-pass filtered from 1 to 35 Hz and the ground velocity was converted to ground acceleration through a differentiation of the signal, then each template component was correlated separately with the correspondent component of the continuous data stream. The effect of the signal processing on both the seismogram and the spectrogram of one of the first located events of the cluster (*Ml* = − 0.2 of 2017-01-21 at 17:25:48 UTC time) is displayed in Supplementary Fig. S6: above 35 Hz the spectral amplitude of the signal starts to become comparable with the noise level principally caused by the attenuation which limits observed peak frequencies^42^; the combined effect of the 1–35 Hz bandpass filter (the lower limit is the minimum frequency of the template length) and the differentiation allows amplifying the peak frequencies of the signal with respect to the noise. To obtain consistent detections for a particular event and to skip detection of distant earthquakes, the maximum time gap was set to 2 s between detections on vertical and horizontals, and to 1 s between horizontals. Finally, the event detection was declared if the result fulfilled all mentioned conditions and occurred at least on two components out of three (see Supplementary Fig. S7 online).

The single-station template matching algorithm applied to the continuous data stream of station INS1 using the selected master events of Supplementary Table S1 allowed the detection of 257 seismic events embedded in the continuous records during the period of investigation from 2016-10-12 to 2018-08-31. Furthermore, from 2018-04-14 to 2018-06-29 the station INS1 suffered a significant disturbance in the seismic signal together with several switch offs due to a current instability. Possible recurrence of this problem was prevented by connecting this station to a power system based on solar panels and batteries^18^. Only in this switch off period the single-station template matching algorithm was applied additionally to the continuous data stream of station INS3, allowing the detection of further 21 events. Therefore, the total number of detected events becomes 278 (224 belonging to the seismicity cluster and additional 54 outliers).

### Event location and magnitude estimation

P- and S-wave first arrival manual picks of waveforms recorded by the stations of the INSIEME seismic network and the Italian National seismic network^47^ for all the detected events were used to perform their absolute locations using the equal differential time (EDT) method implemented in a nonlinear global approach algorithm (NonLinLoc)^25^ and the 3D P- and S-wave velocity model of the area proposed by Serlenga and Stabile^43^. After a first location, a second iteration of location algorithm has been performed with the use of station corrections obtained in the first step. Subsequently, the locations were refined by applying the double-difference method (hypoDD^24^ code, version 2.1), starting from the hypocentral parameters determined from the absolute locations and solving the double-difference equations in the same 3D P- and S-wave velocity model used for absolute locations. Only events with a maximum hypocentral separation of 5 km (*MAXSEP* parameter) and with a minimum number of 5 P- and S-wave linked differential time observations (*MINLINK* parameter) were relocated. Relative locations are performed by linking each event to maximum 30 other events of the cluster (*MAXNGH* parameter) thus obtaining a total number of 25,998 differential times of P- and S-waves. The singular value decomposition (SVD) factorization implemented in hypoDD^24^ has been used which adequately represents least squares errors by computing proper covariances^24^. In the last column of the supplementary seismic catalogue (file “DataS1.csv”) the events only detected (28 events) are labelled as “det”, the absolute located events (26 events) are labelled as “abs”, and the events relocated with the double-difference technique (170 events) are labelled as “rel” (which stands for relative location).

Local magnitude $Ml_{i}$ of each *i*-th located event has been estimated by using the local magnitude scale defined for southern Italy by Bobbio et al.^62^ and applying the Huber mean estimator^63^ $\psi$ to the set of single-station magnitude values $Ml_{ij}$ as follows:

4 $$Ml_{i} = \psi \left( {Ml_{ij} } \right) = \psi \left( {\log A_{ij} + 1.79\log R_{ij} - 0.58} \right),$$

with $A_{ij}$ the peak displacement (in mm) measured from the signal of the *i*-th event recorded by the *j*-th station convolved for the response function of the Wood-Anderson seismograph, and $R_{ij}$ the hypocentral distance (in km) of the *j*-th station from the *i*-th event.

Finally, the moment magnitude $Mw_{i}$ of each *i*-th located event has been computed from the local magnitude estimates $Ml_{i}$ by applying the relationship obtained by Zollo et al.^64^ as follows:

5 $$Mw_{i} = 0.74\left( { \pm 0.01} \right)Ml_{i} + 0.66\left( { \pm 0.02} \right).$$

It allows the estimation of seismic moment $Mo_{i}$ of the *i*-th event through the relationship^65^:

6 $$Mo_{i} = 10^{{\left( {1.5Mw_{i} + 9.1} \right)}} .$$

## Supplementary Information

Supplementary Information 1.

Supplementary Information 2.

## Data Availability

Data of the INSIEME temporary seismic network^18,45^ are open access under the license CC BY 4.0 and they are available from IRIS DMC FDSN Web Services (https://service.iris.edu, last access: April 2021). Information of fluid-injection operational parameters (daily wellhead injected wastewater volumes and daily average wellhead injection pressures) from 2016-09-01 to 2018-08-31 has been provided by the municipality of Montemurro where the CM2 injection well is located. Information on acidification operations (carried out on 17 September 2017) and on the pressure drop along the pipe of the injection well (3 MPa) has been provided by the Eni company.
