# Supplementary material for: Analysis of the 2016–2018 fluid-injection induced seismicity in the High Agri Valley (Southern Italy) from improved detections using template matching
Source: Sci Rep. 2021 Oct 19;11:20630. doi: 10.1038/s41598-021-00047-6 (PMC8526624; doi:10.1038/s41598-021-00047-6)
Supplement: Supplementary file 1 — Supplementary Information 1. [file 41598_2021_47_MOESM1_ESM.pdf]

Analysis of the 2016-2018 fluid-injection induced seismicity in the High Agri Valley (Southern Italy) from improved detections using template matching

Supplementary information

T. A. Stabile[1,*], Josef Vlček[2], Milosz Wcisło[2,3], Vincenzo Serlenga[1]

[1]National Research Council, Institute of Methodologies for Environmental Analysis, Italy
[2]Institute of Rock Structure and Mechanics, Czech Academy of Sciences, Czech Republic
[3]Faculty of Mathematics and Physics, Charles University, Czech Republic

* Corresponding author: tony.stabile@imaa.cnr.it

Mathematical derivation of equation (3) used to analytically compute the minimum and maximum $\boldsymbol{\beta}$ angles of critically stressed fractures for given values of $\boldsymbol{\mu}$, $\boldsymbol{\sigma_1}$, and $\boldsymbol{\sigma_3}$

Indicating with β the angle between the fault normal and maximum principal stress σ_1, the shear stress τ and the effective normal stress σ_n acting on each fracture can be expressed in terms of β, σ_1 and σ_3 (the least principal stress) as follows:

$$\tau = \frac{\sigma_1-\sigma_3}{2}\sin 2\beta, \tag{S1}$$

$$\sigma_n = \frac{\sigma_1+\sigma_3}{2} + \frac{\sigma_1-\sigma_3}{2}\cos 2\beta. \tag{S2}$$

Considering the Mohr-Coulomb failure criterion

$$\tau = \mu\sigma_n \tag{S3}$$

it is possible to substitute τ and σ_n in Equation (S3) with the respective expressions given in Equations (S1) and (S2) to compute the minimum and maximum β angles that satisfies Equation (S3), thus giving:

$$\frac{\sigma_1-\sigma_3}{2}\sin 2\beta = \mu\left(\frac{\sigma_1+\sigma_3}{2} + \frac{\sigma_1-\sigma_3}{2}\cos 2\beta\right) \tag{S4}$$

that can be written, after simple algebra, as:

$$\sin 2\beta - \mu\cos 2\beta - k\mu = 0, \tag{S5}$$

where $k = (\sigma_1+\sigma_3)/(\sigma_1-\sigma_3)$.

Using the following parametric representation of $\sin 2\beta$ and $\cos 2\beta$:

$$\sin 2\beta = \frac{2t}{1+t^2}, \tag{S6}$$

$$\cos 2\beta = \frac{1-t^2}{1+t^2}, \tag{S7}$$

where $t = \tan\beta$ (with $\beta \neq \pi/2$), Equation (S5) becomes:

$$\frac{2t}{1+t^2} - \mu\left(\frac{1-t^2}{1+t^2}\right) - k\mu = 0. \tag{S8}$$

Multiplying all the members of Equation (S8) for $1 + t^2$, after simple algebra it becomes the following quadratic equation:

$$\mu(1-k)t^2 + 2t - \mu(1+k) = 0 \qquad \text{(S9)}$$

whose solutions for t are:

$$t_{1,2} = \frac{-1 \pm \sqrt{1+\mu^2(1-k^2)}}{\mu(1-k)}. \qquad \text{(S10)}$$

Considering that $t = \tan\beta$, Equation (S10) becomes:

$$\beta_{1,2} = \tan^{-1}\left[\frac{-1 \pm \sqrt{1+\mu^2(1-k^2)}}{\mu(1-k)}\right], \qquad \text{(S11)}$$

which is the equation (3).

Description of the Supplementary seismic catalogue (file "DataS1.csv")

List of the 196 located and the 28 detected events belonging to the Costa Molina 2 fluid-injection seismicity cluster analyzed in this study. The seismic catalogue is included in a CSV file with each row reporting the following source parameters: event ID (only for located events); event date (year-month-day); event origin time for located events or event detection time for detected events (UTC time); latitude (˚N); longitude (˚E); depth (km); local magnitude *Ml*; moment magnitude *Mw*; seismic moment *Mo* (N m); additional notes "note". In particular, in the last column "note" the events only detected are labelled as "det", the absolute located events are labelled as "abs", and the events relocated with the double-difference technique are labelled as "rel". Information on event location (latitude, longitude, depth), magnitude estimation (*Ml*, *Mw*), and seismic moment *Mo*, are obviously provided only for located events.

Supplementary Figures

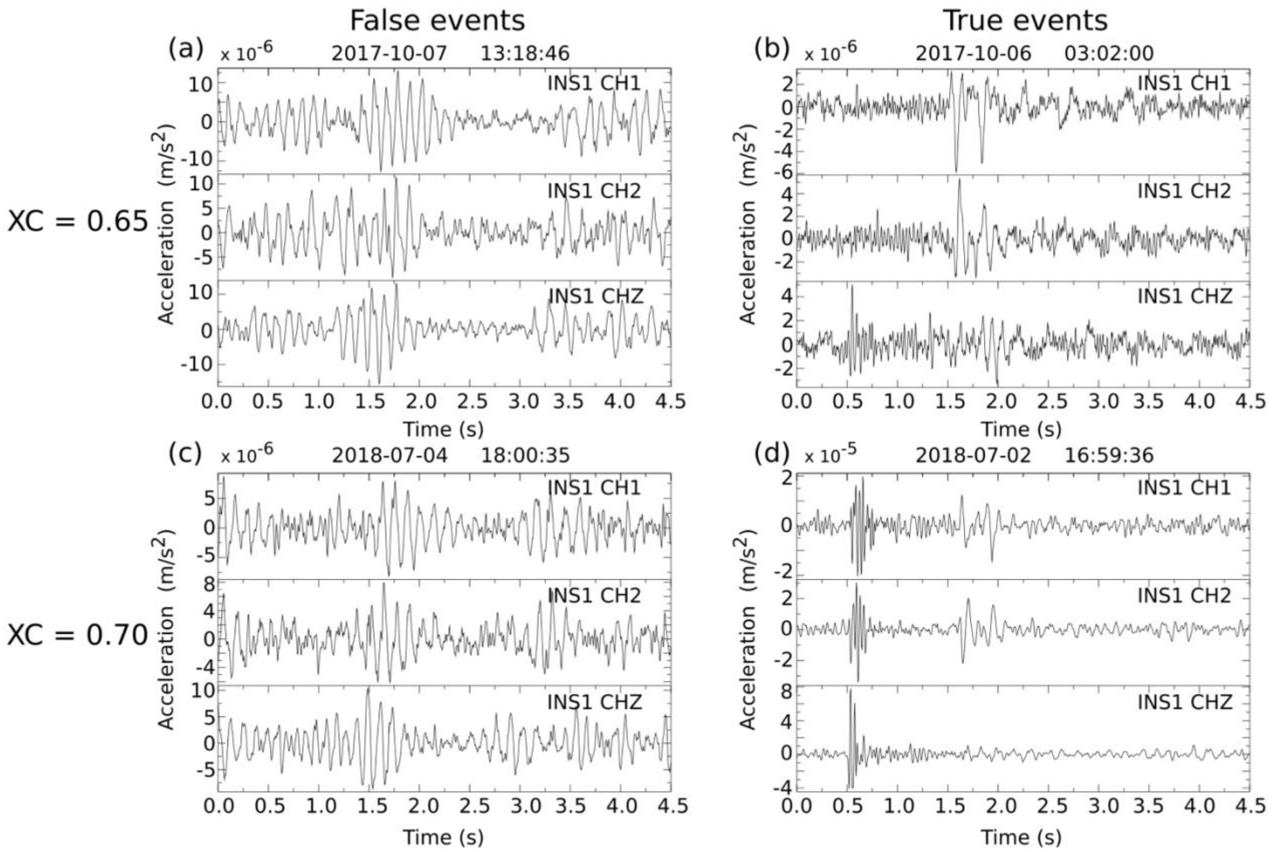

Supplementary Figure S1. Visual comparison of the accelerometric records at the INS1 station related to two couples of false and true events detected by the algorithm adopted in this study. Each chosen couple consists of events characterized by the same value of cross-correlation coefficient (*XC*) and, furthermore, occurred in two days very close to each other. The time appearing in the title of each plot refers to the detection time and a window starting 0.5 s before and ending 4.0 s after the detection time has been represented. a) False event detected at 13:18:46 of 2017-10-07 with an *XC* value of 0.65. b) True event detected at 03:02:00 of 2017-10-06 with an *XC* value of 0.65. c) False event detected at 18:00:35 of 2018-07-04 with an *XC* value of 0.70. d) True event detected at 16:59:36 of 2018-07-02 with an *XC* value of 0.70.

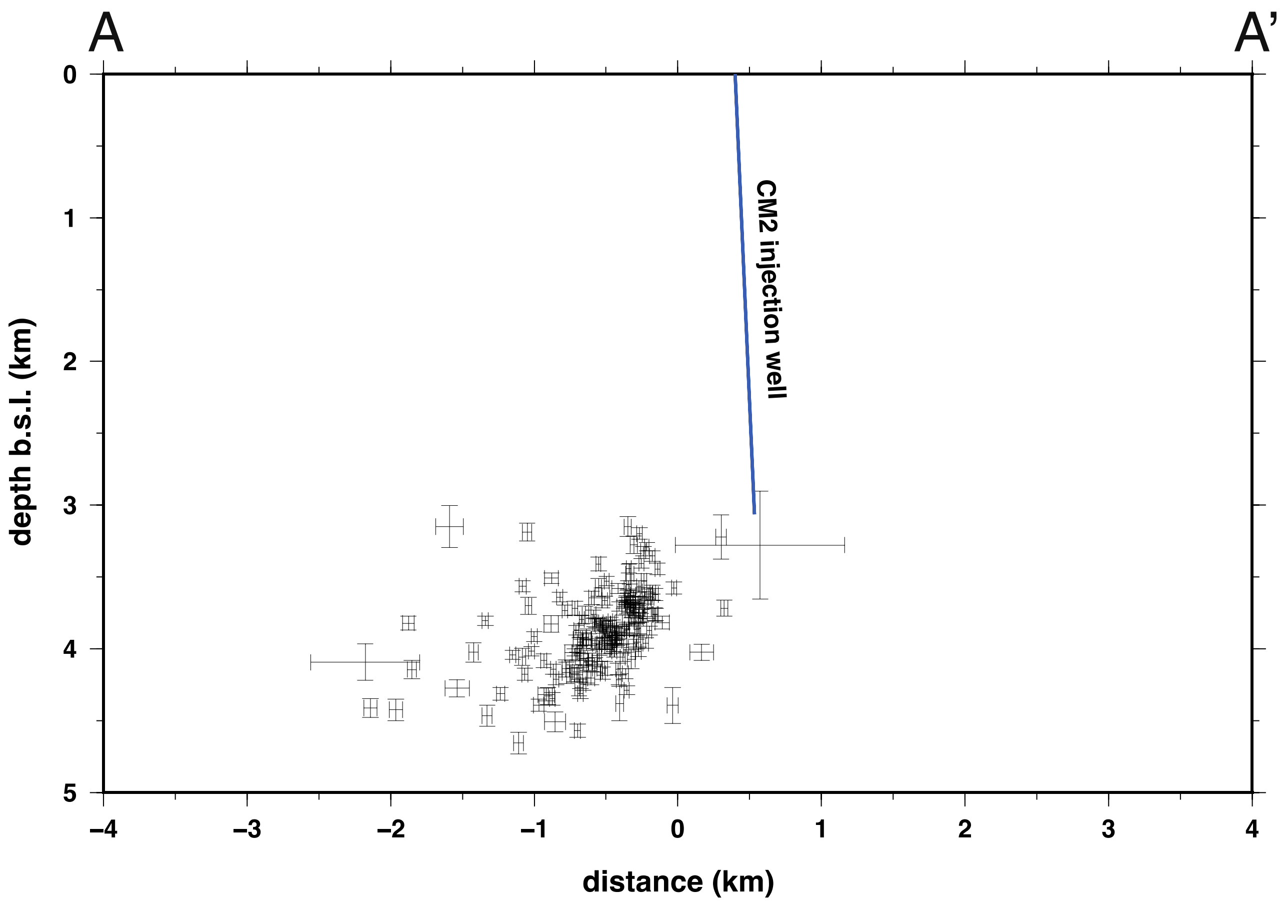

Supplementary Figure S2. Projection of microearthquake hypocenters (only events relocated with double-difference method) along the vertical cross section of the AA' profile displayed in Fig. 1 with their horizontal and vertical relative location errors.

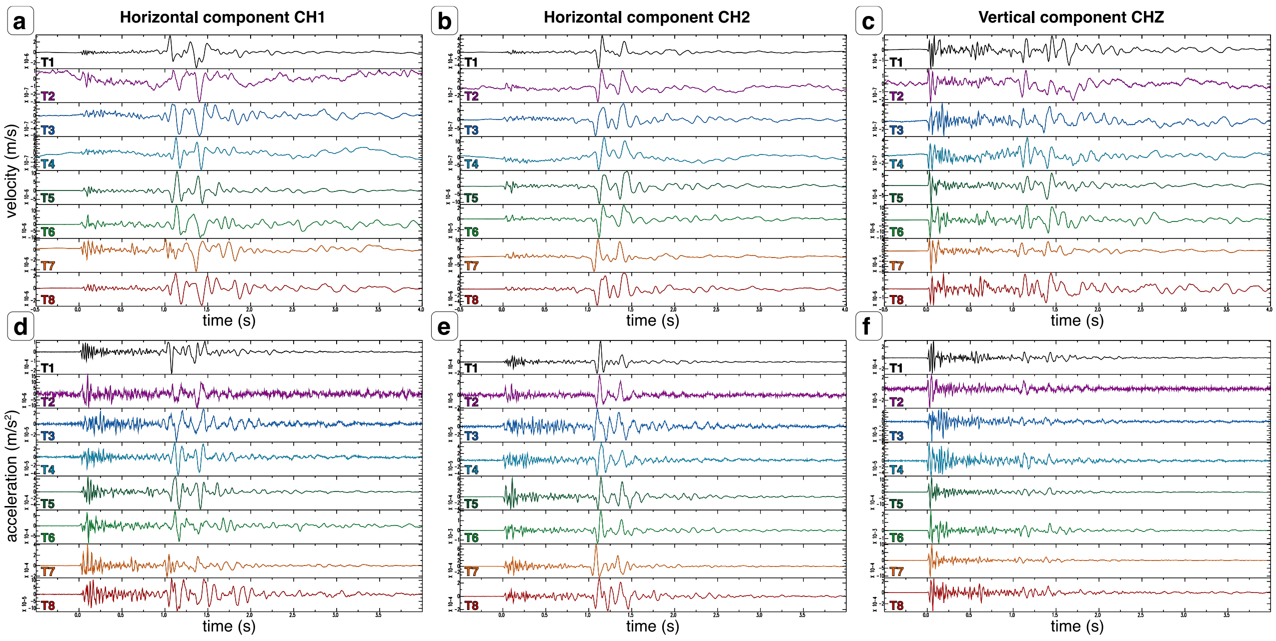

Supplementary Figure S3. Velocity (panels a, b, and c) and acceleration (panels d, e, and f) triaxial components of records at the INS1 station of the eight master events T1-T8 used for the template matching detections. The waveforms are deconvolved from the instrument response, are band-pass-filtered from 0.5 to 80 Hz and are aligned with respect to the first P-wave arrival time.

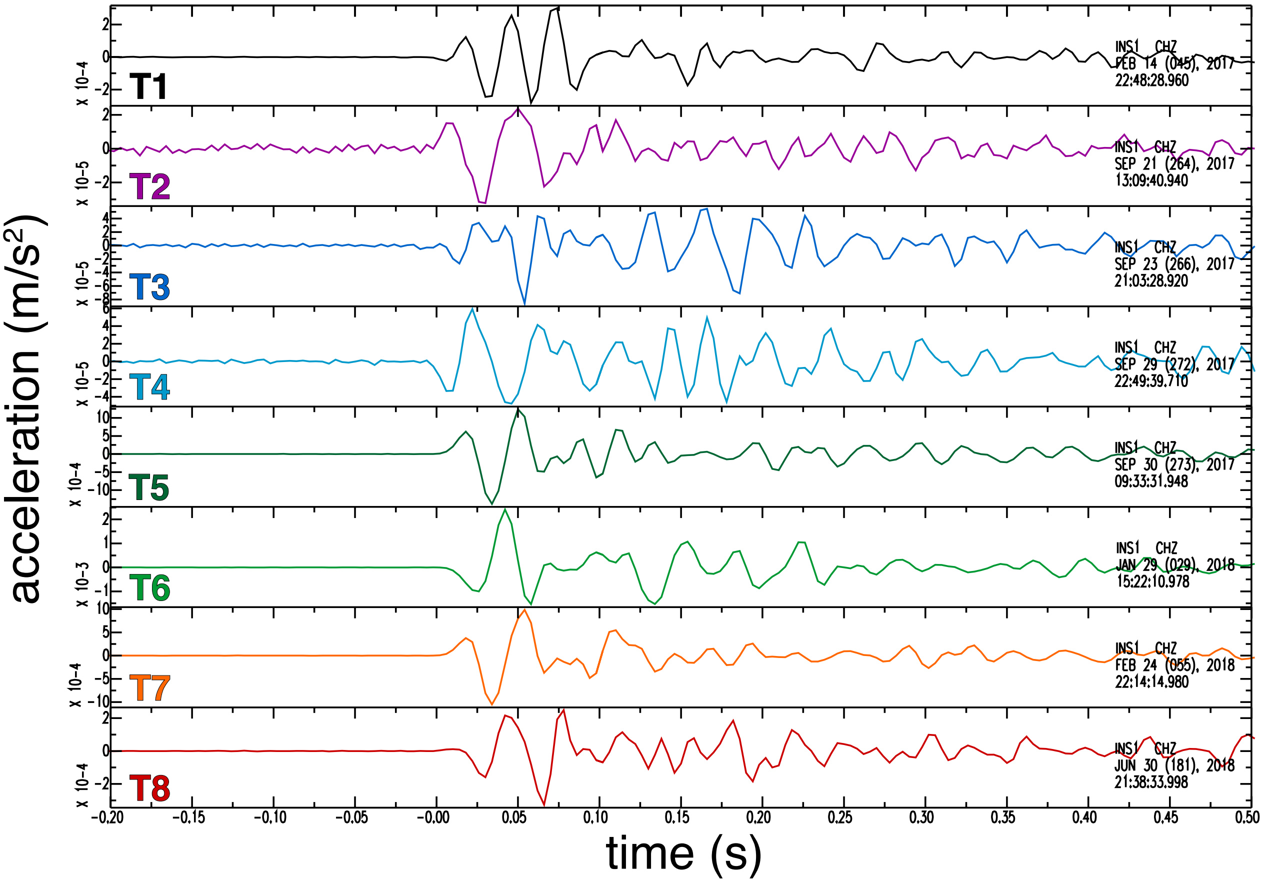

Supplementary Figure S4. Zoom around the P-wave arrival of the ground acceleration vertical components (panel f of the supplementary Fig. S3) of the eight master events T1-T8.

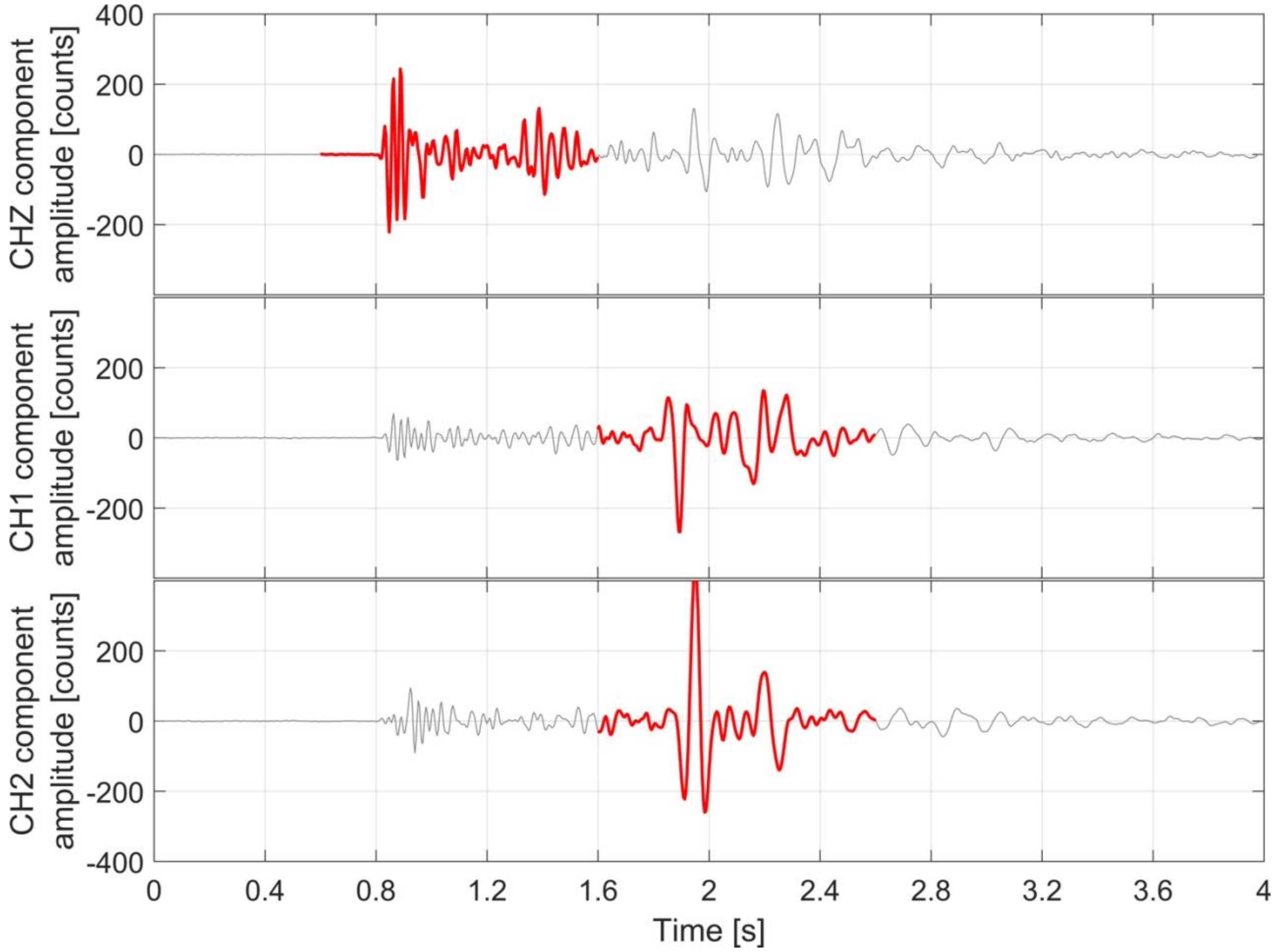

Supplementary Figure S5. Example of one master event waveform (grey) with indication of its selected parts for template detection (red).

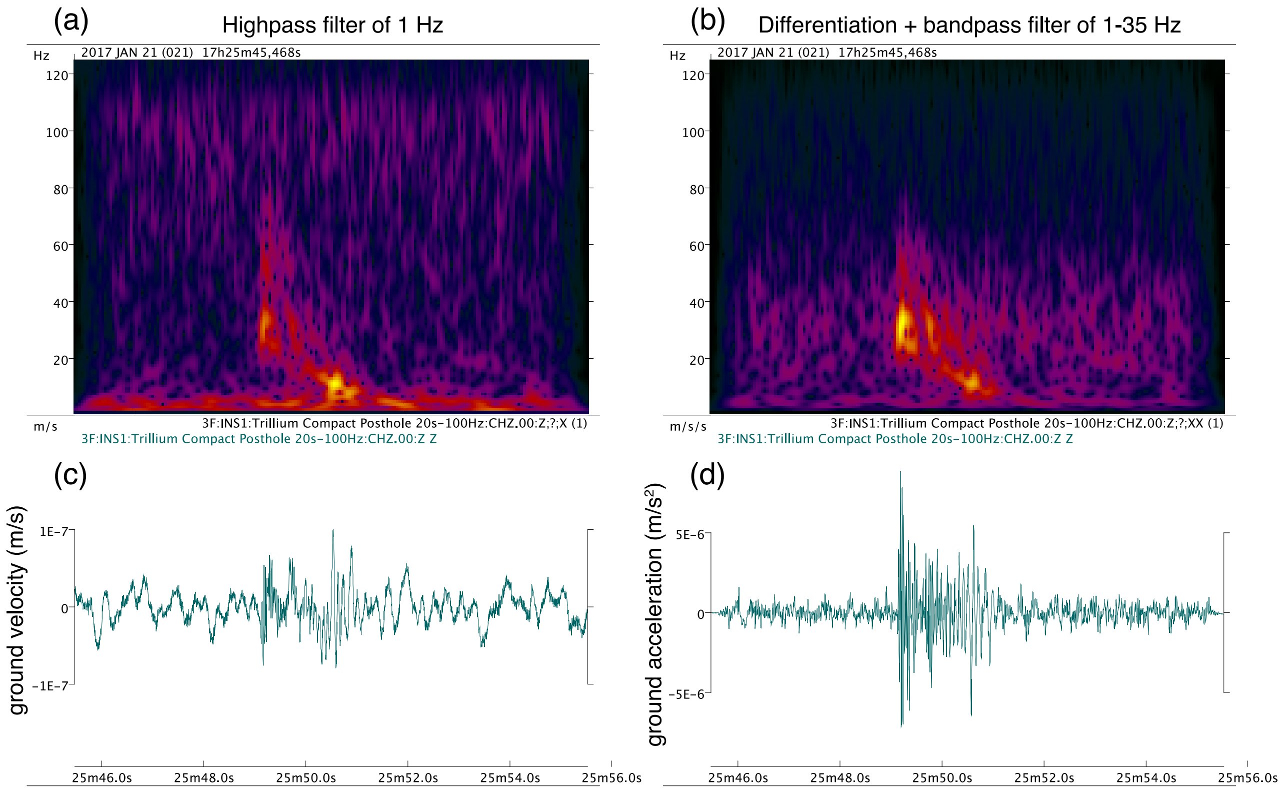

Supplementary Figure S6. The effect of the signal processing on both the seismogram and the spectrogram of one of the first located events of the cluster (event Ml = -0.2 of 2017-01-21 at 17:25:48 UTC time). Spectrogram (panel a) and ground velocity (panel c) of the vertical component of the signal by applying only a highpass filter of 1 Hz. Spectrogram (panel b) and ground acceleration (panel d) of the signal after the application of the differential operator and the 1-35 Hz bandpass filter. The combined effect of the 1-35 Hz bandpass filter (the lower limit is the minimum frequency of the template length) and the differentiation allows to amplify the peak frequencies of the signal with respect to the noise.

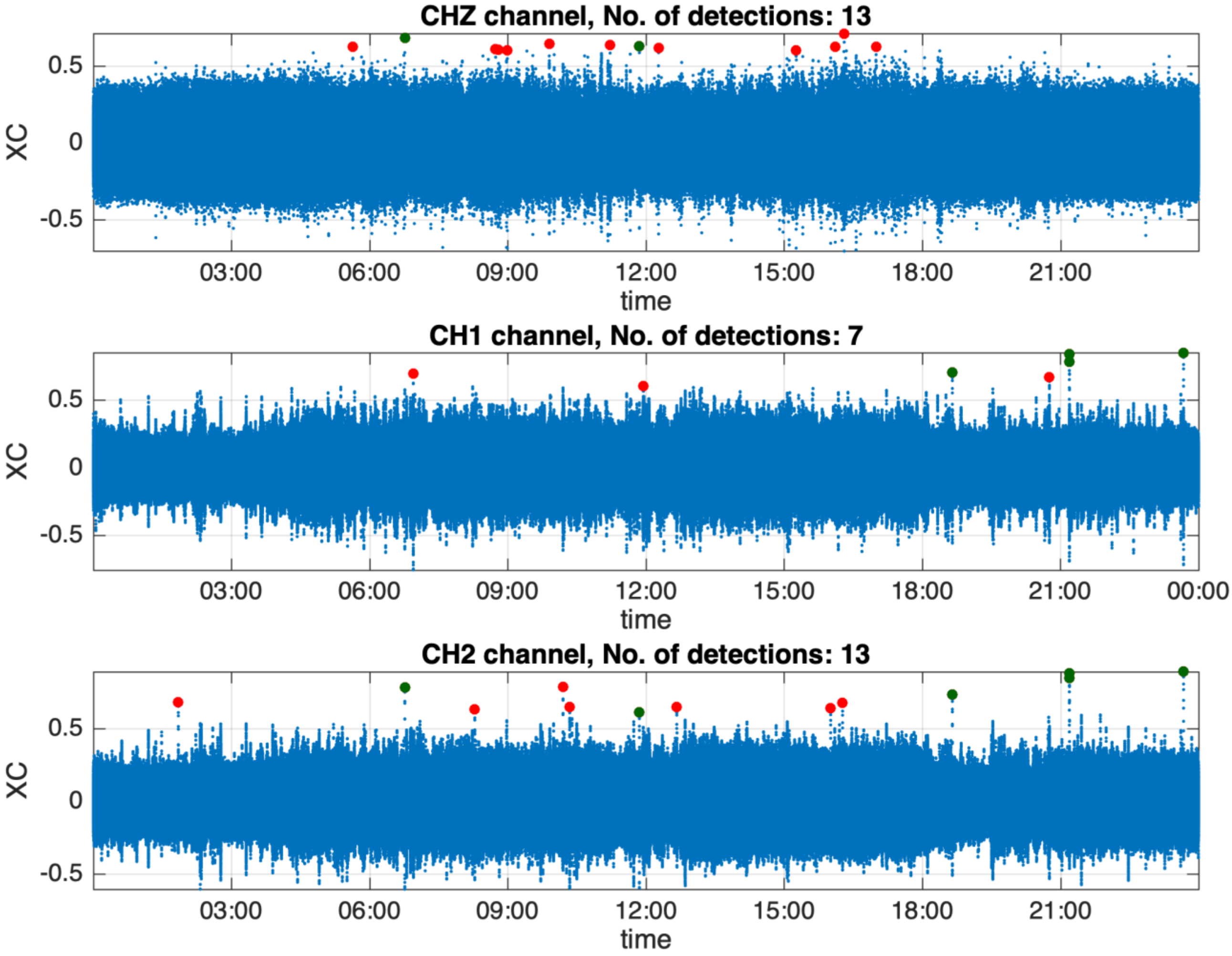

Supplementary Figure S7. Example of cross-correlation coefficients (*XC*) computed over one day of continuous data streams acquired on the three components (CHZ, CH1, and CH2) of station INS1 using the master event template T1. Red circles represent the total number of detections ($XC \geq 0.6$) obtained for each component, whereas green circles represent the declared detections (detection at least on two of the components within the fixed maximum time gap).

Supplementary Tables

Master template name	Date	Origin time	Magnitude Ml
Template 1 (T1)	2017-02-14	22:49:28	0.3
Template 2 (T2)	2017-09-21	13:10:40	-0.3
Template 3 (T3)	2017-09-23	21:04:27	-0.1
Template 4 (T4)	2017-09-29	22:47:40	-0.1
Template 5 (T5)	2017-09-30	09:34:31	0.8
Template 6 (T6)	2018-01-29	15:23:10	1.2
Template 7 (T7)	2018-02-24	22:15:15	0.5
Template 8 (T8)	2018-06-30	21:39:33	0.5

Supplementary Table S1. List of the master event templates selected in this study for detections with the single-station template matching algorithm.

Master template name	No. of detections	No. of true detections	No. of dominant detections	No. of exclusive detections
T1	79	36 (45.6%)	30 (38.0%)	20 (25.3%)
T2	135	97 (71.9%)	33 (24.4%)	12 (8.9%)
T3	92	22 (23.9%)	9 (9.8%)	5 (5.4%)
T4	286	113 (39.5%)	51 (17.8%)	12 (4.2%)
T5	384	135 (35.2%)	61 (15.9%)	32 (8.3%)
T6	49	30 (61.2%)	16 (32.7%)	6 (12.2%)
T7	1452	97 (6.7%)	44 (3.0%)	17 (1.2%)
T8	81	15 (18.5%)	13 (16.0%)	2 (2.5%)

Supplementary Table S2. Basic statistics about detections obtained with cross-correlation for each masters template using station INS1, reporting from the 2nd to the 5th column (in parenthesis the percentages out of the respective total): the number of total detections; the number of true detections; the number of true detections with the highest value of the cross-correlation among all masters that detected given event; the number of events that were detected only by given master.

No. of masters that detected given event	No. of events	% of events
1	101	39.3
2	74	28.8
3	46	17.9
4	24	9.3
5	8	3.1
6	3	1.2
7	1	0.4

Supplementary Table S3. Number and share of events detected by given amount of masters.

Formation	Depth from (m)	Depth to (m)	Layer width (m)	Minimum density (kg/m^3)	Maximum density (kg/m^3)
Albidona Flysch	0	1282	1282	2310	2480
Galestri formation	1282	1526	244	2330	2520
Scisti silicei formation	1526	1616	90	2330	2650
Calcare con selce formation	1616	2097	481	2630	2750
Monte Facito formation	2097	2540	443	2430	2720
Irpine unit	2540	3751	1211	2400	2650
Apulian unit (lower Pliocene)	3751	3899	148	2500	2550
Apulian Platform carbonates	3899	4135	236	2750	2760

Supplementary Table S4. Formations encountered by the Costa Molina 2 well from the wellhead on the surface to the bottom-hole. Minimum and maximum density values of each formation are also reported. Data are provided by Eni Company.
